# Waste-derived volatile fatty acid production and ammonium removal from it by ion exchange process with natural zeolite

**DOI:** 10.1080/21655979.2022.2109507

**Published:** 2022-10-17

**Authors:** Tugba Sapmaz, Amir Mahboubi, Mustafa N. Taher, Bilsen Beler-Baykal, Ahmet Karagunduz, Mohammad J. Taherzadeh, Derya Y. Koseoglu-Imer

**Affiliations:** a Department of Environmental Sciences and Engineering, Istanbul Technical University, Istanbul, Turkey; b Swedish Centre for Resource Recovery, University of Borås, Borås, Sweden; c Department of Environmental Engineering, Gebze Technical University, Kocaeli, Turkey

**Keywords:** Ammonium removal, ion exchange, clinoptilolite, volatile fatty acids, anaerobic immersed membrane bioreactors, resource recovery, waste valorization

## Abstract

Volatile fatty acids (VFAs) produced during anaerobic digestion (AD) of organic waste are a promising alternative carbon source for various biological processes; however, their applications are limited due to the presence of impurities such as ammonium (NH_4_^+^). This study investigates the potential for removal of ammonium using a naturally occurring zeolite (clinoptilolite) from chicken manure (CKM) derived VFA effluent recovered from an anaerobic membrane bioreactor (MBR). Experiments were conducted for both synthetic and actual VFA (AD-VFA) solutions, and the effects of different parameters were investigated with batch and continuous studies. It was observed that the Langmuir-type isotherm provided the best fit to the equilibrium data in the isotherm investigations carried out with the AD-VFA solution. The maximum adsorption capacity (q_m_) was found as 15.7 mg NH_4_^+^/g clinoptilolite. The effect of some operational parameters on process performance such as pH, initial NH_4_^+^ loading and potassium ion (K^+^) concentration was investigated. The pH had a negligible effect on ammonium removal for a pH range of 3–7, while the removal efficiency of ammonium decreased with the increase of initial NH_4_^+^ loading and K^+^ concentration. At the optimum conditions determined in batch experiments, the ammonium removal from synthetic and AD-VFA solutions were compared and average ammonium removal efficiencies of 93 and 94% were found in 12 h equilibrium time for synthetic and AD-VFA solutions, respectively. Overall findings indicated that clinoptilolite has excellent potential for ion exchange when combined with biological processes such as acidogenic fermentation of VFAs to purify the solution from high-ammonium content.

## GRAPHICAL ABSTRACT

**Figure:**
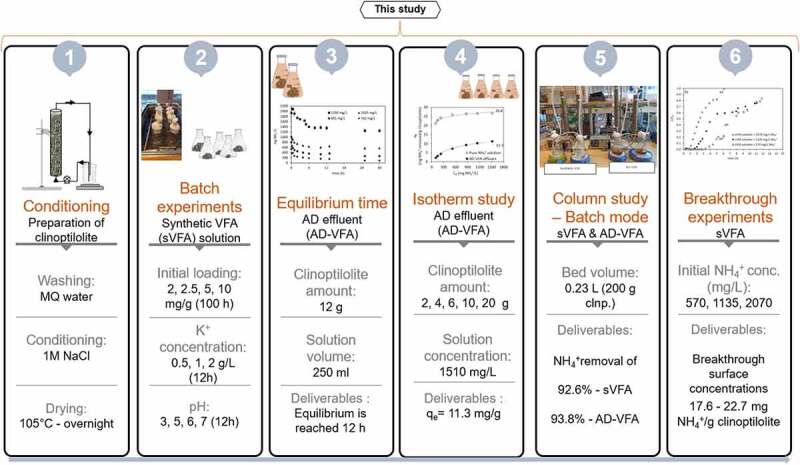

## Highlights

• High NH_4_^+^ content limits the application of CKM-derived AD-VFA (0.90 g VFA/g CKM VS)

• Ion exchange was investigated for the removal of ammonium from VFA mixture

• Targeted ammonium removal was achieved using Na conditioned-form clinoptilolite

• Removal of ammonium through ion exchange yielded high quality VFA_mix_

• An average 94% ammonium removal from AD-VFA was achieved

## 1. Introduction

In order to meet the Sustainable Development Goals and prevent global warming, transmission from a linear to circular economy and conversion of waste back to resources should be implemented [1]. One way to realize the abovementioned goals is to generate highly recovered and sought-after chemicals and materials from waste rather than fossil sources. Volatile fatty acids (VFAs) are sought-after platform chemicals such as acetic, propionic, and butyric acids with great market interest that have conventionally been synthesized from fossil resources. However, these acids are intermediates of microbial degradation of organic wastes, so they can be obtained from anaerobic digestion (AD) of organic wastes and thus contribute greatly to a sustainable circular economy. The acid-forming bacteria in AD processes, which include acetogenic bacteria and homoacetogenic bacteria, are the group of microbes that are primarily in charge of producing VFAs [2]. These VFAs represent a group of valuable carboxylic acids used for a variety of applications, including bioenergy production [3–5], biodegradable plastics [6,7], an additional carbon source for enhanced biological nitrogen and phosphorus removal from wastewater [8–10]. In addition, VFAs can also be used as food additives and animal feed supplements [11,12].

Anaerobic digestion, a common bioconversion approach used mainly for treating organic waste, is a promising option for producing VFAs from renewable and cheap resources in a sustainable manner. AD uses organic-rich waste streams as input material and traditionally converts them to the final product of biogas through a sequential microbial conversion process involving hydrolysis, acidogenesis, acetogenesis, and methanogenesis. VFAs are intermediate products of AD (generated during the acido- and acetogenesis stages) with a higher market value than biogas, which needs to be efficiently produced, recovered, and purified from AD effluent in order to be commercialized. The purer VFA solution has a higher market price and a wider range of applications from production of pharmaceuticals to wastewater treatment [13]. In-depth studies on traditional pre-treatment and co-fermentation methods have also been conducted in the literature with the aim of increasing VFA production and composition, as well as the effects of some key variables that influence VFA composition and production [14]. However, the recovery process of VFAs still poses a great challenge because the AD effluent is a complex medium containing microorganisms, particulate matter, macromolecules, and dissolved nutrients such as nitrogen (N), potassium (K), and phosphorus (P) [15]. Some separation techniques such as precipitation, distillation, adsorption, ion exchange, liquid-liquid extraction, and/or membrane processes can also be used to separate microorganisms and particles as well as macromolecules such as proteins and lipids from AD effluents [16]. Among the newly developed separation technologies for VFA recovery from anaerobic digestate, membrane-based processes are considered to be very attractive and competent methods [16].

The high ammonia content in the AD effluents is one of the challenges in building a VFA platform. Considering the potential use of VFAs as an additional carbon source for wastewater denitrification process, or the production of polyhydroxyalkanoates (PHAs) as bioplastics, the NH_4_^+^ content of the effluent is an undeniable drawback. Although the presence of a certain amount of ammonium is beneficial for microbial growth in AD, inhibitory concentrations can be reached during the degradation of protein-rich substrates. Therefore, in processes where limited nitrogen concentrations are important, the excess amount of ammonium in the VFA-containing AD effluent should be removed with minimal loss of VFAs. Common technologies used for ammonium removal include breakpoint chlorination, air stripping, nitrification-denitrification, and ion exchange [17]. Among these methods, ion exchange has gained great research and industrial attention due to process simplicity and the application of low-cost abundant raw minerals as exchangers [18]. For this purpose, natural zeolites such as clinoptilolite could be applied as the ion-exchanging material. The high selectivity of clinoptilolite for ammonium ion is an asset and gives it advantage over conventional and synthetic exchange resins such as purolite and amberlite [19].

Ion exchange is a special type of adsorption based on electrostatic attraction. In this process, the pollutant (adsorbate) or compound to be removed is separated from the liquid phase and concentrated on the solid phase (adsorbent). Ion exchange can also be considered as a reversible chemical reaction driven by electrostatic attraction between the ions in solution (adsorbate) and the exchangeable ion on the ion exchanger [20]. There are many studies using the ion exchange process to recover nutrients from different types of waste streams [21–24]. However, to the knowledge of the authors, the removal of ammonium from the organic waste derived AD-VFA effluent with clinoptilolite has not been studied. Therefore, it is of great importance to understand the ion exchange performance of clinoptilolite for the removal of ammonium from VFAs mixtures. This is an important application since production of VFAs and removing contaminants like ammonium may be key technology in the upcoming biorefineries. It is essential to carry out an experimental study to assess the different effects of the various processes on the functionality of VFA production and recovery to comprehend how this can be done and which operational elements influence the recovery of purer and higher concentrated VFAs from fermentation broths.

The objective of this study is to investigate a well-established process, ion exchange, for a new application, namely the removal of ammonium from bio-based VFAs mixtures. The purpose of this study is to research the removal of ammonium from synthetic and chicken manure derived AD-VFA solutions by ion exchange using natural zeolite (clinoptilolite). Following this, batch tests will be used to determine the effects of initial ammonium loading, pH, and the most common competing ion, K^+^, on ammonium removal. The experimental steps of ion exchange process that were carried out includes isotherm studies, batch-column experiments for ammonium removal, and continuous experiments for producing breakthrough curves.

## 2. Materials and methods

### 2.1. Chemicals

Acetic acid (>99.7%), propionic acid (>99.5%), butyric acid (>99%), NH_4_Cl, and NaCl were purchased from Sigma-Aldrich (Burlington, MA, US) and KCl of reagent grade was provided from Merck (Darmstadt, Germany). The water used in the preparation of all synthetic solutions was ultrapure (Milli-Q at 25°C).

### 2.2. The solid phase: clinoptilolite

The natural zeolite, clinoptilolite, which is an alumina silicate was used as the ion exchanger. It was obtained from Rota Mining Corporation and originated from the mines of the Gordes region in Manisa, Turkey. Its mineralogical composition was determined by Rota Mining [25] as 90–95% clinoptilolite, 0–5% cristobalite, and 0–5% tridymite. The shared oxygen atoms between aluminum and silicon in clinoptilolite result in a negative charge that is balanced by the presence of exchangeable cations such as Ca^2+^, K^+^, and Na^+^, depending on the composition. The chemical formula of clinoptilolite is (Ca, K_2_, Na_2_, Mg)_4_ Al_8_ Si_40_ O_96_.24H_2_O with chemical composition as shown in Table 1 [25]. Its theoretical exchange capacity was 1.5–2.1 meq/g based on the mineralogical composition according to Methylene Blue Chloride Method as indicated in the product information sheet by Rota Mining Corporation [25]. The apparent density of the clinoptilolite was found to be 0.86 g/cm^3^ using the standard method. Clinoptilolite was firstly sieved to obtain a specific particle size, which in this study was 1–2 mm. The sieved clinoptilolite was then washed several times with distilled water to remove non-adherent impurities and small particles, followed by air-drying for 24 h to remove moisture. It was proven in the literature that the sodium form of clinoptilolite is the most recommended form to provide the highest capacity for the removal of ammonium, therefore, Na-form of clinoptilolite was used for the experiments [26]. Clinoptilolite has a monoclinic platy crystal structure with angular granules as its physical appearance [25]. The utilized particle size ranged from 1000 to 2000 μm. The conditioning of clinoptilolite aims to remove certain ions from the surface to replace them with easily exchangeable ions prior to ion exchange application. Clinoptilolite in the sodium form is known to have a higher ammonium removal capacity as compared to its original form [26–29], therefore, using the sodium form is beneficial [30]. For this reason, prior to the ion exchange process, conditioning was carried out to increase the ammonium holding capacity of clinoptilolite. Using a 1 M NaCl solution according to the procedure recommended by Yurtoğlu [26], at flow rate of 11.7 L/min for up to 48 h. Prior to use in experiments, the clinoptilolite was dried at 103–105°C to remove moisture in an attempt to obtain constant weight. The same batch of conditioned clinoptilolite was used in all runs of the experimental work.

**Table 1.** Characteristics of the clinoptilolite (from Rota [25]).

Constituent	Properties/ Value (wt.%)
SiO_2_	65–72
Al_2_O_3_	10–12
CaO	2.4–3.7
K_2_O	2.5–3.8
Fe_2_O_3_	0.7–1.9
MgO	0.9–1.2
Na_2_O	0–0.08
MnO	0–0.08
Cr_2_O_3_	0–0.01
P_2_O_5_	0.02–0.03

### 2.3. Liquid phase

Two different solutions were used: waste-derived anaerobic digestion VFA effluent (AD-VFA) and synthetic VFA solution (sVFA).

#### 2.3.1. Anaerobic digestion VFA effluent (AD-VFA)

Chicken manure (CKM) was used as AD substrate and inoculum was collected from a laying hen farm Sjömarkens Hönsgård AB (Borås, Sweden) with total solid (TS) and volatile solid (VS) concentrations of about 320 and 220 g/L, respectively. To ensure consistency, as-received CKM was diluted with tap water to VS of about 120 g/L (~2 times dilution) and mixed with a high-performance blender (Waring® CB15, CT, USA) at high speed for about 30s. The liquid phase was separated with a sieve (mesh size about 0.1 mm) and the characteristics were checked after thorough mixing. Thermal pretreatment (thermal shock) was used to inhibit the activity of methanogens in CKM. Each feed solution was placed in a 100 mL of flask, then heated to 80°C for 15 min in a water bath while being completely mixed at a speed of approximately 300 rpm to ensure a homogeneous temperature, and then cooled in an ice chamber. Some properties of the sieved thermally shocked CKM (the feed for AD) are pH of 7.9 ± 0.1, total VFA concentration of 4.5 ± 0.2 g/L, of which acetic acid content of 3.8 ± 0.2 g/L, and ammonium concentration of 4500 mg/L. The supernatant from the heat shocked CKM (mesh size approximately 0.1 mm) was diluted to approximately 10 g VS /L and used as inoculum for the production of VFAs effluent in an anaerobically digested membrane bioreactor (MBR). The operation of MBRs is described in more detail in Section 2.4.1. The VFA solution filtered from the reactor each day was collected and stored in the freezer before it was used further in experiments. AD-VFA solution that is mixed and stored had a pH of 6.8, ammonium of 1955 ± 135 mg/L and total VFA concentration of 7 ± 0.2 g/L (Table 2). AD-VFA solution was used in the isotherm studies and the batch mode fixed bed column operation during this study.

**Table 2.** The properties of recovered AD-VFA.

Parameters	Unit	Range of the effluent during fermentation	VFA effluent used in this study
pH	/	6.02–8.18	6.8 ± 0.1
NH_4_-N	mg/L	1381–2262	1955 ± 135
tCOD	g/L	12.0–68.7	13.8 ± 0.6
Acetic acid	g/L	5.0–11.9	5.37 ± 0.2
Propionic acid	g/L	0.8–3.1	0.81 ± 0.1
Butyric acid	g/L	0.6–2.4	0.75 ± 0.1
Caproic acid	g/L	0.05–0.15	0.07 ± 0.0
Total VFAs	g/L	6.50–18.0	7.0 ± 0.2
Na^+^	mg/L	986.3–1045.7	1016 ± 29.7
K^+^	mg/L	1535.6–1572.4	1554 ± 18.4
Ca^2+^	mg/L	32–33	33 ± 0.0
Mg^2+^	mg/L	59.4–63.6	61.5 ± 2.1
Fe^2+^	mg/L	1.8–3.2	2.5 ± 0.7

#### 2.3.2. Synthetic VFA solution (sVFA)

The synthetic VFAs solution was prepared by dissolving acetic acid, propionic acid, butyric acid, and NH_4_Cl in Milli-Q water to represent VFAs and ammonium content. The total concentration of VFAs was arranged as 7.1 g/L theoretically to simulate waste-derived VFA effluent (AD-VFA) content including acetic, propionic, butyric acids at the ratio of 4.5:2:0.6, respectively. pH of the solution was measured as 2.8 ± 0.2 and the amount of NH_4_^+^ was changed in the range of 500–2070 mg/L. In order to study the effect of K^+^ ion on process performance, KCl solution was prepared as 0.5 and 2 M for required K^+^ ion concentration. Synthetic VFA solution was used in batch experiments (the effect of initial loading, pH, and K^+^ concentration), batch mode fixed bed column operation and breakthrough experiments to understand the system behavior of processes investigated.

### 2.4. The experimental setup

#### 2.4.1. Experimental set up and operation of MBRs for AD-VFA production

**Figure 1.**
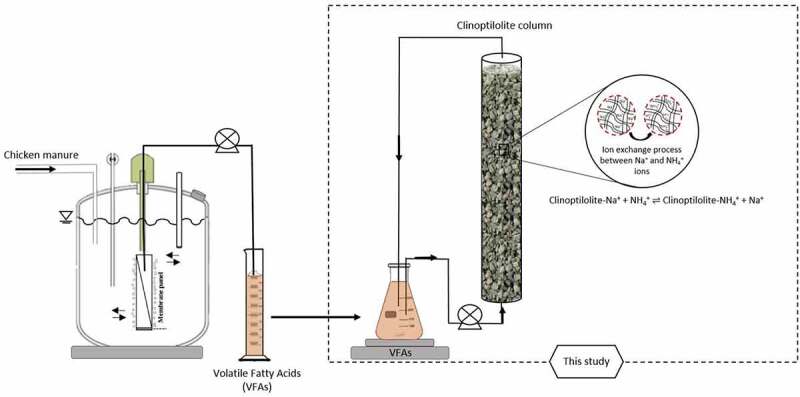
Schematic overview of MBR setup for AD-VFA production and fixed bed batch column setup for ammonium removal.

In order to generate the VFAs effluent required for this study, MBR using CKM as feed was used. The MBRs that were operated semi-continuously (Figure 1) used for the anaerobic digestion of CKM consisted of a reactor with a 2^nd^ generation microfiltration integrated permeate channel membrane panel (PES, 0.3 μm average pore size; VITO NV, Belgium), with a filtration area of 205.8 cm^2^ submerged in a reactor (Biotech GmbH, Germany) and with 3.5 L working volume. Permeate flow and membrane pressure were recorded automatically using a control recording system developed in-house. pH, the concentrations of VFAs, and ammonium concentrations from permeate in the reactor were recorded by daily. The 350 mL of permeate containing VFA was filtered off daily and kept in freezer before use in ion exchange experiment.

#### 2.4.2. Isotherm experiments

Waste-derived AD-VFA was used in the isotherm experiments. In order to generate the isotherms for ammonium removal by clinoptilolite, the equilibrium time was determined first using AD-VFA solutions with concentrations of 1930, 1035, 801, 524 mg NH_4_^+^/L prepared by serial dilution of AD-VFA. After that, the isotherm experiments were carried out to determine the capacity of clinoptilolite for ammonium in AD-VFA mixture in duplicates (Equation 1) [31].

(1) $$Equilibrium\;capacity\left(mg\;N{H_{4}}^{+}/g\;clinoptilolite\right),\left(q_{e}\right)=\frac{(C_{0}-C_{e)}\times V}{m}\;$$

C_0_ is the initial ammonium concentration (mg/L), C_e_ is the equilibrium concentration of ammonium in the solution (mg/L), V is the volume of the solution (L), m is the mass of the clinoptilolite used (g), q_e_ is the amount of adsorbate per mass of the adsorbent under equilibrium conditions (mg/g). During the isotherm experiments, 2.0, 4.0, 6.0, 10, and 20 g clinoptilolite was put in each flask including 50 mL of AD-VFA solution having 1955 ± 135 mg/L of ammonium placed in a water bath-shaker (Grant OLS 200, Grant instrument ltd, UK) for 12 h which had been determined as the equilibrium time. The ammonium concentrations of the samples were measured at the beginning of experiment and then at the equilibrium time (12 h). All experiments were performed at temperature of 22 ± 2°C. Isotherms were plotted as the amount of ammonium adsorbed onto the clinoptilolite, *q_e_*, against the equilibrium concentration of ammonium in the solution, *C_e_*. The experimental results were fitted to Langmuir Isotherm model (Equation 2) and Freundlich model (Equation 3) to determine the best isotherm model that represented the experimental data.

(Eq.2) $$q_{e}=q_{m}K_{L}\frac{C_{e}}{1+K_{L}C_{e}}$$

(Eq.3) $$q_{e}=K_{F}{C_{e}}^{1/n}$$

In these non-linear expression of isotherm models, K_L_ is a Langmuir constant associated with adsorption energy or net enthalpy, K_F_ is the Freundlich constant and n is a constant with a function of adsorption intensity and q_m_ is the surface concentration obtained when all adsorption sites are exhausted (maximum capacity of adsorbent) [32].

#### 2.4.3. The effects of pH, initial ammonium loading and competing cation (K^+^) in batch experiments

Batch experiments were performed as duplicate to investigate the influence of pH (3, 5, 6, and 7), initial ammonium loading (2, 2.5, 5.0, and 10 mg NH_4_^+^/ g clinoptilolite), and competing ion-K^+^ concentration (500, 1000, and 2000 mg/L) using a synthetically prepared VFA solution. The experiments were carried out in the batch mode using conical Erlenmeyer flasks of 250 mL (working volume of 200 mL) placed in a temperature-controlled water bath shaker at 110 rpm and with a temperature of 22 ± 1°C. The aforementioned synthetic VFAs mixture (Section 2.3.2) was used at this stage.

The effect of pH on the removal of ammonium by the ion exchange process with clinoptilolite has been studied in the literature [33]. In this study, since the removal of ammonium was studied in the presence of VFAs, and the tests were carried out at different pH values for observing the changing of VFA species with pH changes. The pKa values of the targeted VFAs are reported as 4.76 for acetic acid, 4.88 for propionic acid, and 4.82 for butyric acid. The pH values of AD-VFA solutions may change based on the substrate type and the operational parameters of the anaerobic digester. The pH values of the synthetic VFAs mixture and AD-VFA solution were measured as 2.8 ± 0.2 and AD-VFA and 6.8 ± 0.1, respectively. Thus, the effect of pH on overall ion exchange performance was investigated at 3.0, 5.0, 6.0 and 7.0. The ion selectivity of clinoptilolite was given by the manufacturer as Cs^+^ > NH_4_^+^ > Pb^2+^ > K^+^ > Na^+^ > Ca^2+^ > Mg^2+^ > Ba^2+^ > Cu^2+^ > Zn^2+^ [25]. In the experiments of competing ion effect, different K^+^ concentrations in the range of 500 to 2000 mg/L were tested due to high concentration of potassium ion (K^+^: 1554 ± 18.4 mg/L) that could compete with the ammonium ion strongly in the exchange process (Table 2).

The effect of initial ammonium loading (mg NH_4_^+^ initially loaded per gram of clinoptilolite) was studied in the batch mode by changing clinoptilolite loading through the use of constant ammonium (760.50 ± 54.83 mg/L) and VFAs concentrations (7.46 ± 0.51 g/L) and variable amounts of clinoptilolite. All the experiments were carried out at the same stirring rate and in duplicates.

#### 2.4.4. Batch fixed bed column experiments

In this part of experiments, the ion exchange experiments for the removal of ammonium from two different solutions, synthetic, and AD-VFA solutions were carried out in fixed bed columns. The fixed bed columns were all made of Plexiglas and operated in duplicates. The columns were 3 cm in inner diameter and 35 cm in height (Figure 1). The column experiments were carried out in batch and up-flow mode. Fixed bed column experiments were performed until equilibrium was attained, which had taken at most 30 hours. AD-VFA solution was fed to the column at a flow rate of 28 ± 1.6 mL/min using a peristaltic pump, Watson-Marlow 403 (Watson Marlow, United Kingdom). Bed volume was calculated by measuring the height of the bed and found as 0.23 L when filled with 200 g clinoptilolite for an initial loading 2.5 mg NH_4_^+^/g clinoptilolite. The pH for synthetic VFA was adjusted to the same value as of AD-VFA (pH: 6.8). In this study, two columns were operated using two different solutions and samples were taken at certain time intervals, and the results were given with mean and standard deviation values.

#### 2.4.5. Breakthrough experiments

In this step, breakthrough curves at three different ammonium concentrations were generated for ammonium in synthetically prepared VFA solution, to observe system behavior. Breakthrough experiments were performed in up-flow fixed bed columns with continuous flow mode and at flowrates of 14 ± 1.5 ml/min. Synthetic solutions were prepared with different initial ammonium concentrations (570, 1135 and 2070 mg/L) and a constant VFA concentration of 7.1 ± 0.8 g/L. The height of the bed was kept constant, and the column was filled fully (bed height: 33 cm). Effluent from the column was sampled continuously and the samples were analyzed for ammonium concentrations. The experiments were conducted at 16.6 min empty bed contact time (EBCT) that was found with Equation 4, where V_b_ is the bed volume and Q denotes the flowrate in the fixed bed column [34].

(Eq. 4) $$EBCT=V_{b/}Q$$

### 2.5. Analytical methods

For characterization of VFAs, total solid (TS) and total suspended solid (TSS) contents were measured using oven at 105°C following the standard methods of the American Public Health Association (APHA-AWWA-WEF, 2005). Total chemical oxygen demand (tCOD) was measured using the CSB 15000 test kits (range of 1.0–15.0 g/L O_2_) (Nanocolor, MACHEREY-NAGEL GmbH & Co. KG, Germany) for characterization of AD-VFA. The concentration of tCOD was measured using the Nanocolor 500D photometer (MACHEREY-NAGEL GmbH & Co. KG, Germany). The analysis of trace elements (Fe, Mg, K, Na, and Ca) was performed using microwave plasma atomic emission spectrometry (MP-AES 4200, Agilent Technology, Santa Clara, USA). The pH was measured using a pH combination electrode from Orion Ross Model according to the manufacturer’s recommendations. Ammonium concentration was measured using an Orion 720 ion meter and ammonia electrode. Prior to ammonium measurements and VFA analysis, samples were filtered through a 0.45 μm microporous membrane filter. VFA compositions (acetic, propionic, isobutyric, butyric, isovaleric, valeric acid and caproic acid) were analyzed by means of Perkin-Elmer gas chromatograph (GC) (Clarus 590; Norwalk, CT, USA) equipped with a flame ionization detector (FID) and a capillary column (Elite-WAX ETR, 30 m × 0.32 mm × 1.00 μm, Perkin-Elmer, Shelton, CT, USA). The injection port and the detection temperature were maintained at 250°C and 300°C, respectively. Nitrogen was used as the carrier gas at a flow rate of 2 mL/min and a pressure of 20 psi. Prior to analysis, the liquid samples were mixed with an acid mixture (25% (v/v) formic acid and 25% (v/v) ortho-phosphoric acid in a 1:3 ratio) to pronate the -COOH groups of the VFAs needed to facilitate extraction in the sample, and then centrifuged at 10,000 × g for 5 min. The supernatant was then filtered through a 0.2 μm syringe filter to remove particulate matter. The range of VFA standards used were between 0.3125–10 g/L. Butanol solution with a concentration of 1 g/L was used as an internal standard.

## 3. Results and discussion

Thermally pre-treated CKM was used as substrate in an immersed MBR for enhanced VFA production and in situ recovery of VFAs in a semi-continuous AD. It was aimed both to investigate the production of VFA and to remove NH_4_^+^ from this solution. Batch and continuous ion exchange studies were conducted to investigate ammonium removal from collected AD-VFA effluents by clinoptilolite. Batch tests were used to investigate the effects of some important parameters on ammonium removal, while column studies were used as preliminary studies for the practical application of continuous VFA treatment. Within this context, the effects of initial ammonium loading, presence of K^+^ ions and pH on ammonium removal were investigated in batch tests. Then, ammonium removal from synthetic and real VFA media (AD-VFA) was compared and batch column studies were conducted to investigate the effects of other constituents in AD-VFA on ammonium removal. In the final phase, breakthrough experiments were performed at constant empty bed contact time (EBCT) at different ammonium concentrations and a preliminary evaluation was made for future studies.

VFAs were produced from the fermentation of CKM at organic loading rates of 2 to 4 g VS/L/d. The effluent bearing VFAs that were to be used from ion exchange were obtained by in-situ recovery from anaerobic acid fermentation reactor using an immersed microfilter (unpublished work). The properties of the permeate effluent mixture are presented in Table 2. The organic composition of the substrate as well as the source of the substrate influence the type of the VFAs produced [35]. It was also proposed that operational conditions may dictate which catabolic product would be dominant, allowing for more efficient growth in the mixed culture fermentation system at the same time [36]. The VFA formed after fermentation is not pure, but rather contains a considerable mineral impurity in an aqueous solution [37]. NH_4_^+^-N content in the permeate was in the range of 1381–2262 mg/L during the fermentation of CKM. The daily fermentation effluents from the reactor were homogeneously mixed, and the characteristics of the solution employed in this study were determined (Table 2). Ammonium content has been found to be 1955 ± 135 mg/L (Table 2). K^+^ has the highest concentration (1554.0 ± 18.4) among other cations in the effluent solution (Table 2). The concentration of impurities may vary depending on the source of the wastewater/feedstock and the type and conditions of the fermentation [37]. In the current study, in situ recovery of VFAs-rich filtrate by the MBR not only prevented the washing out of microorganisms, ensuring the continuous and stable operation of the anaerobic acid fermentation process [38,39], but also obtained a VFAs solution with low turbidity [40], suitable for downstream processing such as ion exchange for ammonium removal.

### 3.2. Isotherm experiment results

**Figure 2.**
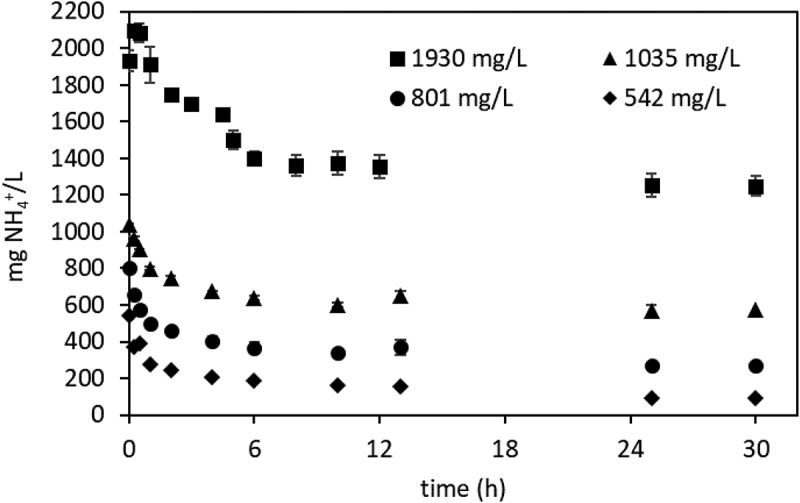
Equilibrium time profile with different initial adsorbate concentrations.

In this study, isotherm experiments were performed in a real VFA solution (AD-VFA), and the isotherm constants and ammonium removal capacity were compared with the isotherm constants obtained in pure ammonium solution in distilled water without other constituents such as competing ions determined by using the same type of clinoptilolite [32]. The objective is to determine how the ammonium removal capacities change in AD-VFA solution. Such a comparison is critical for designing real applications. Isotherms were generated from equilibrium data using clinoptilolite and AD-VFA solution in an attempt to evaluate ammonium removal capacity. For this purpose, firstly, equilibrium time experiments were carried out at four different ammonium concentrations that may be expected to prevail in actual large-scale operation, for 30 hours as can be seen in Figure 2, and 12 hours was chosen as the equilibrium time.

**Figure 3.**
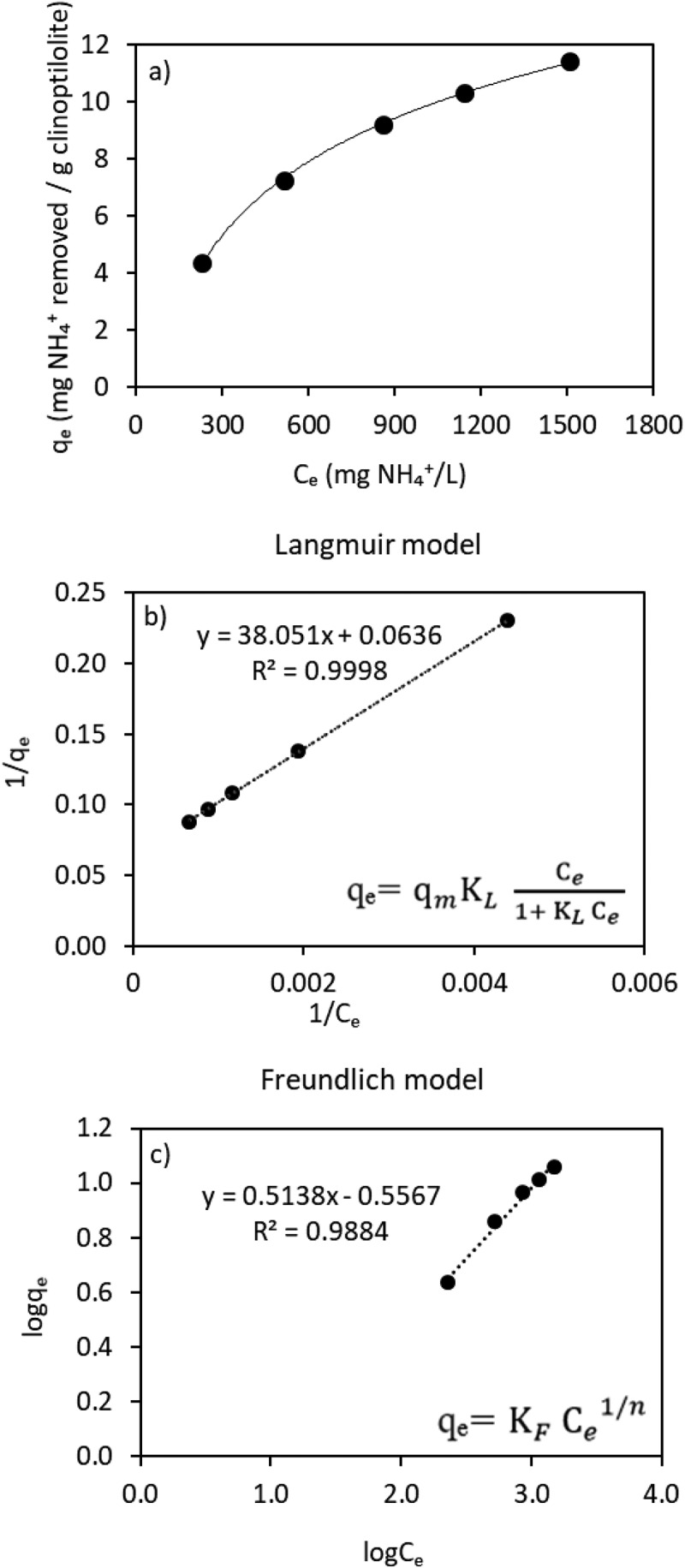
(a) Isotherm curve and (b) Langmuir and (c) Freundlich isotherm models of ammonium with clinoptilolite for AD-VFA solution.

Both the Freundlich and Langmuir models were used to evaluate the experimental results of ammonium removal from a real VFA solution. As can be seen from Table 3 and Figure 3, Langmuir model had a slightly better fit compared to the Freundlich isotherm model, with a regression coefficient (R^2^) value on Langmuir, which is 0.9998. A comparison of the regression coefficients in these two model solutions (pure ammonium and AD-VFA) reveals that the Langmuir model represents the system better than the Freundlich model. In the Langmuir model, an adsorbate is bound to the active adsorption site and all surface areas are assumed to have the same attractive force. According to the Langmuir model, the maximum adsorption capacity (q_m_) was found as 15.7 mg ammonium/g clinoptilolite (Table 3) while the equilibrium capacity (q_e_) shows 11.3 mg/g for 1510 ± 85 mg NH_4_^+^/L equilibrium liquid phase concentration in Figure 3a.

**Table 3.** The values of isotherm model equations.

	Langmuir model				Freundlich model			
	Equation	q_m_	K_L_	R^2^	Equation	K_f_	1/n	R^2^
Pure solution (Allar [41])	$q_{e}=\frac{0.9438Ce}{1+0.0345Ce}$	27.4	0.0345	0.9826	$q_{e}=3.8966{C_{e}}^{0.2356}$	3.8966	0.2356	0.8723
AD-VFA (This study)	$q_{e}=\frac{0.02628Ce}{1+0.0017Ce}$	15.7	0.0017	0.9998	$q_{e}=0.277{C_{e}}^{0.5138}$	0.277	0.5138	0.9884

**Figure 4.**
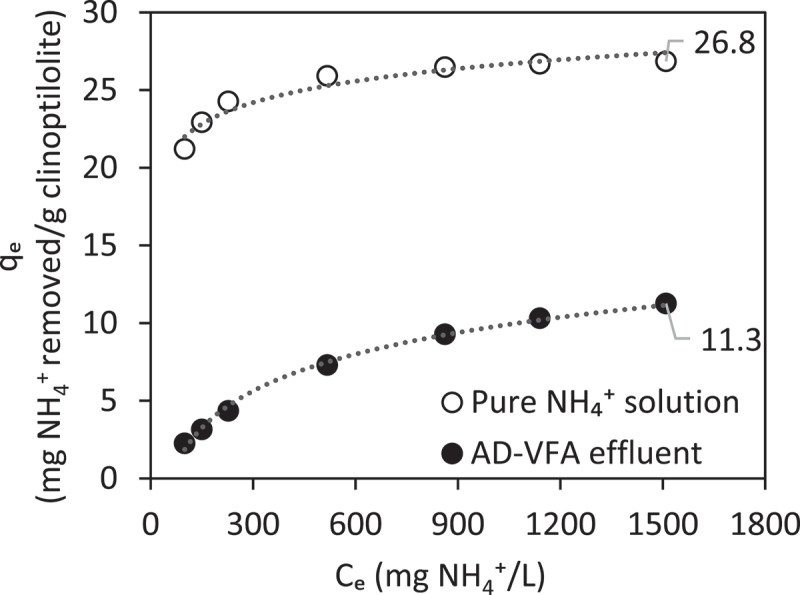
Comparison between ion exchange isotherms of pure NH_4_^+^ solution, and AD-VFA with q_m_ values from Langmuir. The figure contains results from experiments that were done in a previous study done by Allar [41].

The C_e_ values obtained in this study were placed in the Langmuir equation obtained by Allar [41], and while the q_m_ value was 27.4 mg/g, the q_e_ value for 1510 mg/L was found as 26.8 mg/g. The graph for pure solution and AD-VFA comparatively is shown in Figure 4. The difference of equilibrium capacities between the pure solution and the AD-VFA solution is 15.5 mg NH_4_^+^/g. This difference shows that existence of ions other than ammonium in AD-VFA solution has reduced the equilibrium capacity for ammonium for this specific solution. The content and the concentration of competing cations were measured with MP-AES device and their concentrations are presented in Table 2. As shown in Table 2, the average potassium concentration in the real VFA environment was measured as 1554 ± 18.4 mg/L. In addition, the ammonium concentration was measured as 1955 ± 135 mg/L and was very similar to potassium concentration. K^+^ competes with ammonium [42] and hence the ammonium removal by ion exchange of AD-VFA is lower than in pure solution. According to a study done by Rizzioli, Battista [43] on the purification and concentration of VFAs using various batch adsorption tests, Lewatit and Amberlyst ion exchange resins had respective adsorption yields of 40 and 27%. A real fermentate with an initial VFA concentration of roughly 18 g/L was subjected to the best adsorbent, Lewatit, and the best desorption conditions, resulting in a final VFA content that was three times higher than in the original solution [43]. Depending on the source and the fermentation process applied, Na^+^, K^+^, H_2_PO_4_^–^/HPO_4_^2–^, Cl ^–^ and SO_4_^2^^–^ ions are common in fermentation effluents [37]. However, negatively charged ions are not expected to interfere with the cation exchange process and K^+^ might be the most critical competing ion. In addition to these ions, AD-VFA contains other soluble organic compounds as well as VFA [43].

### 3.3. The effect of initial NH_4_^+^ loading on ammonium removal

**Figure 5.**
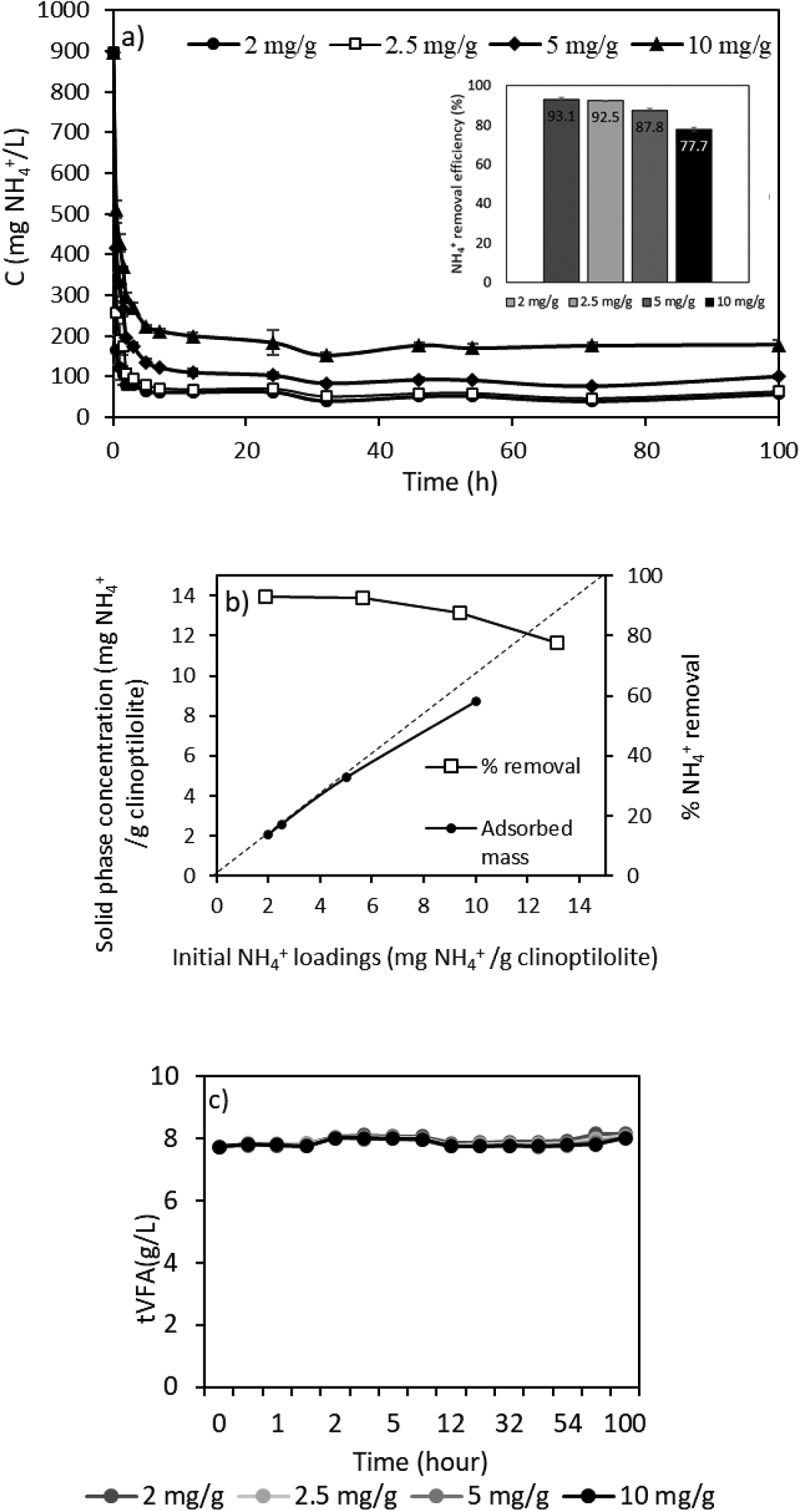
Effect of initial loading on ammonium removal with ion exchange from sVFA solution.

One of the most important design parameters in the ion exchange process is the initial ammonium loading which affects the removal efficiency of the system [44]. The initial ammonium loadings (mass of NH_4_^+^/mass of clinoptilolite) were arranged as 2.0–2.5-5.0–10 mg NH_4_^+^/g clinoptilolite and the graph of ammonium removal is presented in Figure 5a and 5b. The figures show that at the initial ammonium loading of 2.5 mg NH_4_^+^/g clinoptilolite, ammonium removal was found to be over 92%, and higher initial loadings lead to relatively lower efficiencies which will not permit the product to be used effectively, as an external carbon supplement for denitrification for instance. It may be observed that ammonium removal increased with decreasing the initial loadings up to 2.5, whereas further reduction of initial loading down to 2 mg/g did not result in a significant improvement in ammonium removal. Increasing the amount of clinoptilolite leads to lower initial loadings per g of adsorbent within same initial ammonium concentration. The removal of ammonium is essentially unaffected up to the initial loading threshold of 10 mg NH_4_^+^/g clinoptilolite, and a removal efficiency of 94% was achieved for ammonium in a study on the transfer of plant nutrients from urine to clinoptilolite by examining the removal efficiency under various initial ammonium loadings (5–34 mg NH_4_^+^/g clinoptilolite) [44]. Hence, in accordance with previous findings, increasing the amount of adsorbent to a certain extent resulted in increased removal of ammonium ions [44,45]. Therefore, initial ammonium loading, 2.5 mg NH_4_^+^/g clinoptilolite, was selected for the rest of the experiments. There was no VFAs loss from the solution during the sets of initial loading tests (Figure 5c).

### 3.4. The effect of pH

**Figure 6.**
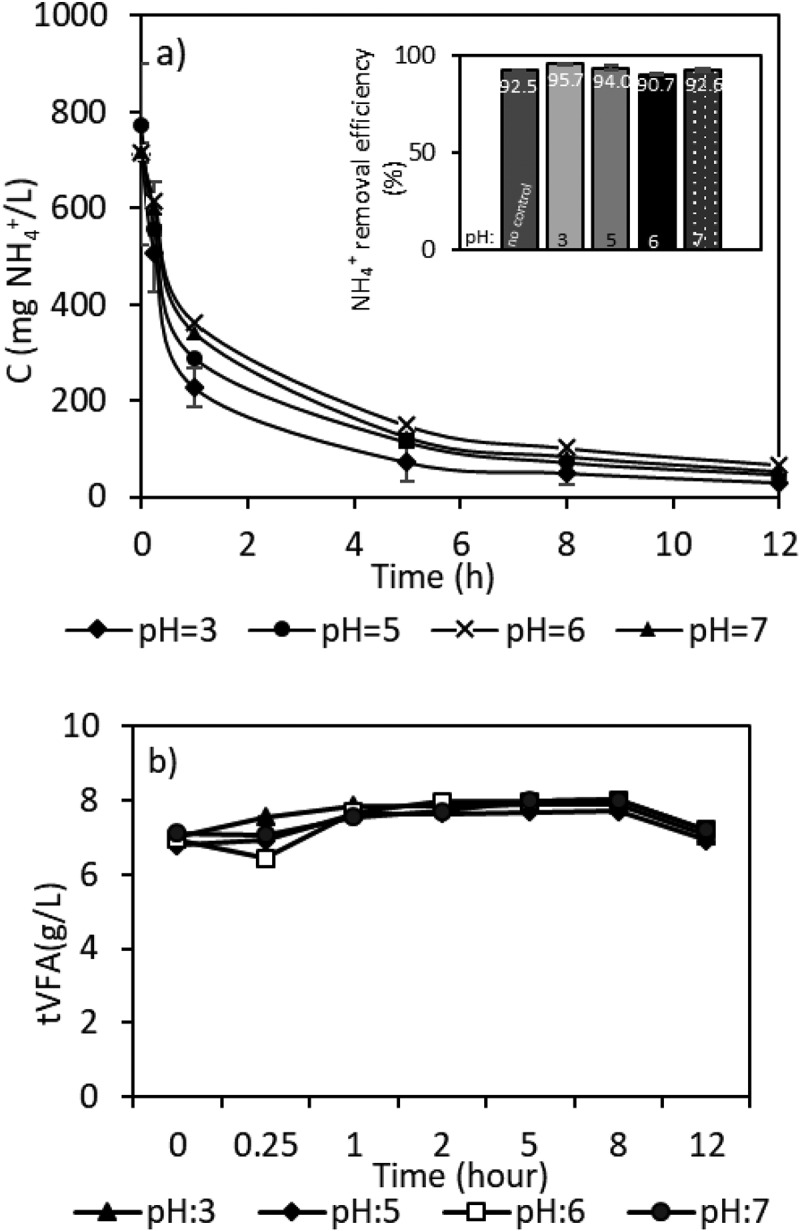
Effect of pH on ammonium removal with ion exchange from sVFA solution.

The pH of solutions was 2.8 ± 0.2 for synthetic VFA and 6.8 ± 0.1 for AD-VFA effluent. The pH is an important factor for ammonium/ammonia equilibrium in ion exchange processes. Although ammonium is the dominant form at low pH, its concentration starts to decreases at pH values above 8 (pK_a_ of ammonium is 9.25) as it transforms to the non-ionized form of ammonia [46] which cannot undergo ion exchange process. From the perspective of ammonium removal, pH values below 8 are not expected to be critical, and that is what the results show in this present work as well, as may be observed in Figure 6a, b. Moreover, whether VFAs exist in dissociated or undissociated (free acid) form depends on pH as well [47], therefore, VFA concentrations were also measured during the ion exchange batch studies. This has led to conducting batch experiments at different pH values ranging from 3–7 to determine the effect of pH on the overall ion exchange process. In this range of pH, the efficiency of ammonium exchange was not directly affected by the change in pH. VFAs exist mainly as undissociated acids at pH values below the dissociation constant of acids (≤ 4.86 at 25°C) [48]; however, the dissociated form of VFAs is dominant in higher pH values [37]. As can be seen in Figure 6a, b, pH had no significant effect on VFAs concentrations as well as ammonium removal. The maximum removal (about 96%) for ammonium was found at pH 3, as shown in Figure 6a moreover, the removal efficiency of ammonium was still above 90% at all different pH values investigated in this work as expected. The results in this study show that pH values between 3 and 7 have no significant effect on the ammonium removal efficiency. It was evident that the presence of VFAs did not affect the removal of ammonium (with a 0.5–1% decrease only). There is nearly no VFAs loss from solution during ammonium adsorption (Figure 6b). Similar results were obtained in a previous study using clinoptilolite for ammonium removal from leachate [33]. In that study, pH values of leachate were in the range of 4 to 10. It was shown that changes in the pH in the range between 6 and 8 had little effect on ammonium removal with the best removal performance was recorded at pH of 7 with 57.8% [33]. Specific anions in the solution could reduce ammonium adsorption due to hydrolyzation of ions and the associated pH change [49], therefore, the VFA concentrations were also controlled during the ion exchange batch studies. As the synthetic solution used has both ammonium and VFAs it was of importance to see the effect of both cations and anions. There are also some studies on the removal of VFAs by anion exchange resins [50–54]; however, clinoptilolite is a cation exchanger. Hence, it does not affect the concentrations of deprotonated VFAs (carboxylate salts) in the effluent.

It can be concluded that addition of chemicals for pH adjustment would not be required when ion exchange system was adapted directly to the fermenter, which would mean that no additional cost would be required. This possible integration of processes eliminates the need to integrate an additional pH adjustment and control system, which indirectly reduces the cost.

### 3.5. The effect of K^+^ concentrations on ammonium removal

**Figure 7.**
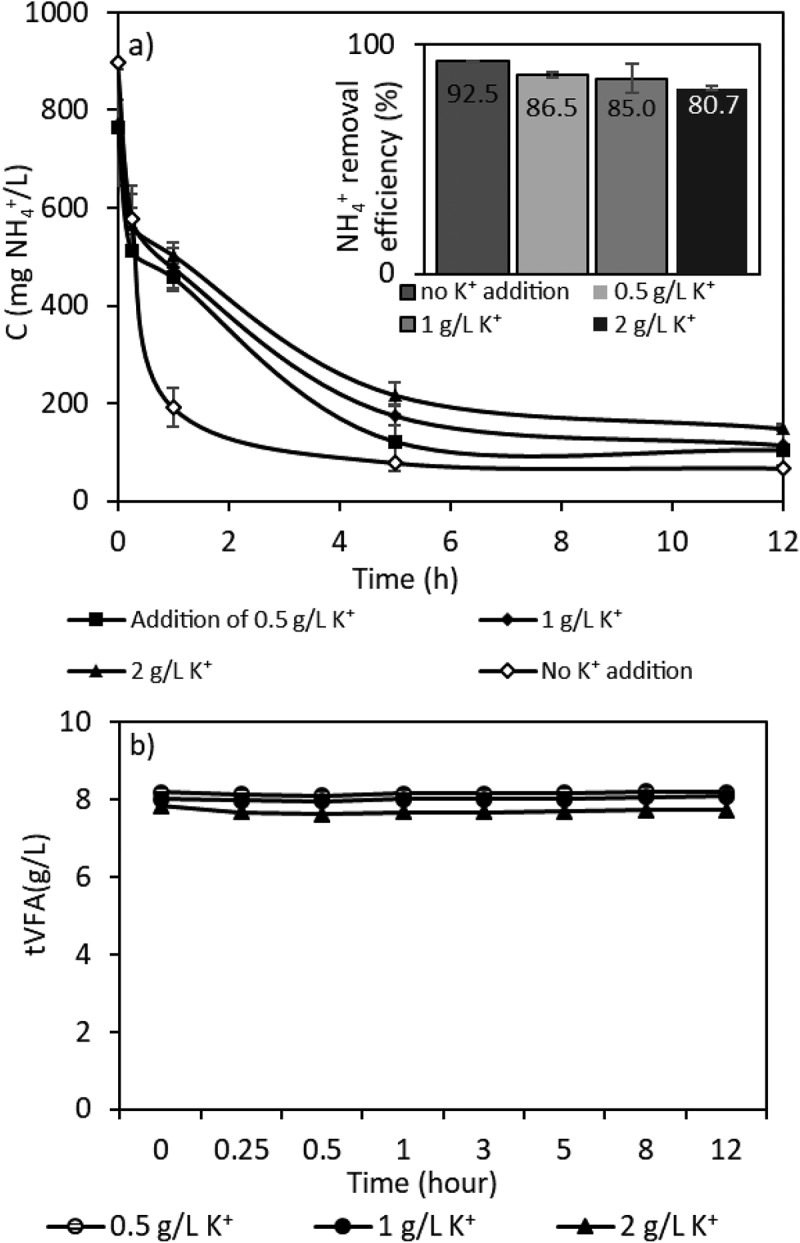
Effect of K^+^ ion on ammonium removal with ion exchange from sVFA solution.

In this part of the study, the effect of potassium ion on ammonium removal was studied since it is a constituent found in higher concentrations in real VFA effluent and has a competing effect on ammonium removal (Table 2). The cations in the solution and ammonium would compete with each other, resulting in a decrease in ammonium exchange by the ion exchanger [55]. The selectivity order of clinoptilolite between cations at similar concentrations is Cs^+^ > NH_4_^+^ > Pb^2+^ > K^+^ > Na^+^ > Ca^2+^ > Mg^2+^ > Ba^2+^ > Cu^2+^, Zn^2+^ as it is given by manufacturer [25]. Although the clinoptilolite used in this study is a highly ammonium-selective zeolite [56], at comparable concentrations of the major competing cations, the increased concentration of K^+^ and Ca^+^ ions could alter the selectivity order of the clinoptilolite depending on the characterization of the fermentation liquid. As shown in Figure 7a, with the addition of maximum 2 g/L K^+^ compared to no addition of K^+^, the removal efficiencies dropped from 92% to 81%, while initial ammonium concentration decreased from 898 mg/L to 67 mg/L and from 773 mg/L to 149 mg/L, respectively.

Wang, Lin [42] showed that the presence of potassium, calcium and magnesium ions would compete with ammonium ions for ammonium exchange with the Chinese Na-modified zeolite. Of these ions, the presence of potassium had the strongest effect on the reduction of ammonium removal, followed by calcium. This is consistent with the results of another study by Farkaš, Rožić [57], where both support the findings of reduction on ammonium removal. There is negligible VFA loss from the solution during the run of different K^+^ ion tests (Figure 7b).

### 3.6. Ammonium removal from synthetic and AD-VFA effluent using fixed bed columns

**Figure 8.**
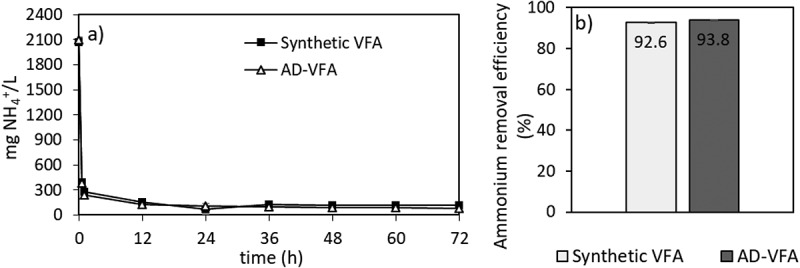
Ammonium removal of clinoptilolite in sVFA and AD-VFA solutions (pH 6.8).

**Figure 9.**
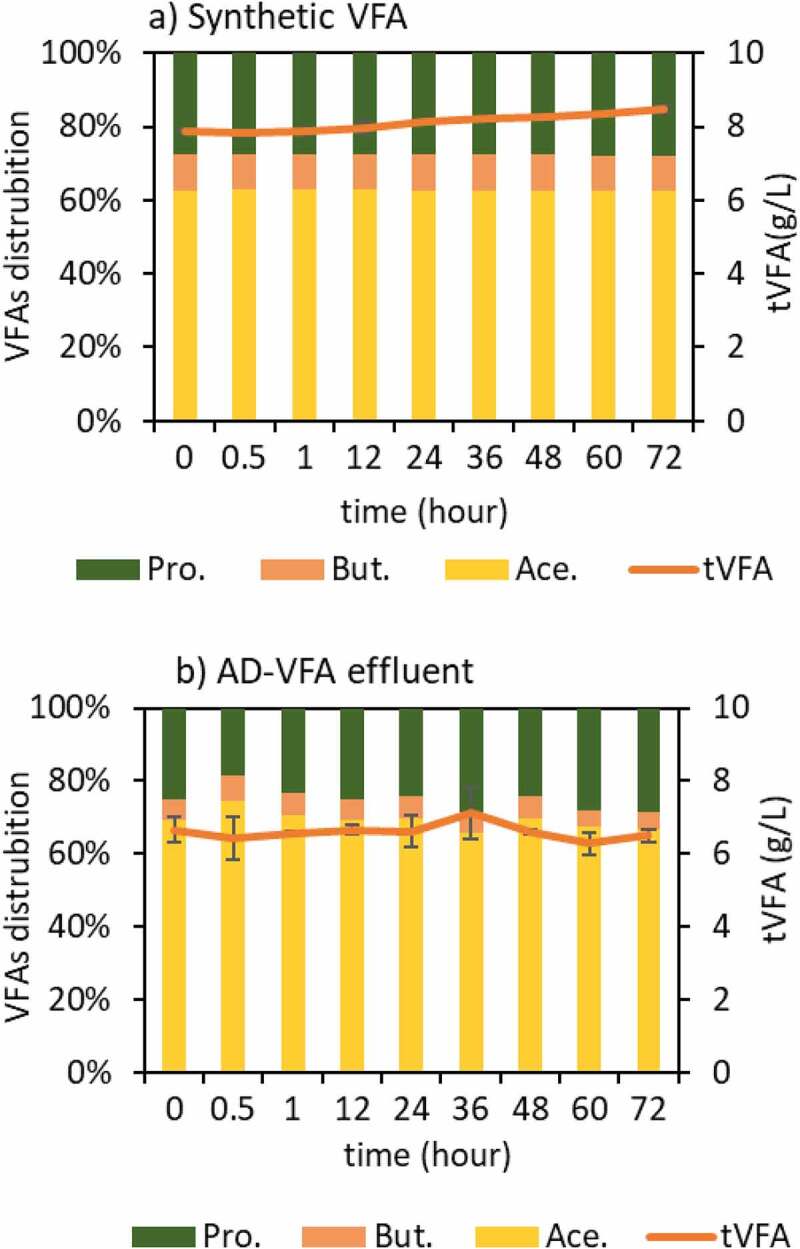
VFA changes on (a) sVFA and (b) waste derived AD-VFA solution.

Based on the results of different initial loadings batch experiments, an initial loading of 2.5 mg NH_4_^+^/g clinoptilolite was selected for full circulation (=batch) column experiments. In the data obtained in the Section 3.1 of isotherm experiments, the equilibrium capacity (q_e_) that is evaluated through Langmuir isotherm was 26.8 mg/g in the pure solution and the capacity decreased to 11.3 mg/g due to the presence of other constituents in AD-VFA medium. In the experiments that is presented in Figure 8, however, there was no difference between synthetic VFA and AD-VFA for ammonium removal. In this experimental stage, the removal of ammonium was investigated in fixed bed column using both AD-VFA and synthetic VFA solution with an initial tVFA concentration of 7.9 ± 0.8 and 5.9 ± 0.3 g/L, and ammonium concentrations of 2090 ± 36 and 2067 ± 41.6 mg NH_4_^+^/L, respectively. For a clearer comparison, ammonium removal and VFAs concentrations were analyzed and presented in Figures 8 and 9, respectively. It can be observed that starting from an initial value around 2100 mg/L ammonium, which could come from the fermentation of protein- and/or urea-rich wastes such as animal manure, especially chicken manure, or slaughterhouse wastes, most of the ammonium content is removed within 12 hours. The results indicated that other ions present in AD-VFA did not decrease the ammonium removal percentages; in fact, the ammonium removal efficiency of 93.8% was almost comparable to that of synthetic VFA (92.6%) at a reaction time of 12 hours (Figure 8b). Therefore, while a slight K^+^ effect was observed in the batch experiments, K^+^ showed no negative effect in these column experiments. Ion exchange process from AD-VFA solution allowed even higher ammonium removals even in the presence of other ions due to its large ion exchange capacity. It can be explained that the concentrations of other ions (K^+^, Na^+^, Mg^2+,^ Ca^2+,^ Fe^2+^) were much lower than the ammonium concentration in real VFA medium and also their selectivity order was lower than that of ammonium. Table 2 shows that the concentration of the competing cation K^+^ in the AD-VFA was 1554 ± 18.4 mg/L, while that of ammonium was 1955 ± 135 mg/L. The composition and the concentrations of the VFAs were stable during the 72-hour experiments (Figure 9).

### 3.7. Preliminary breakthrough experiments for ammonium removal

**Figure 10.**
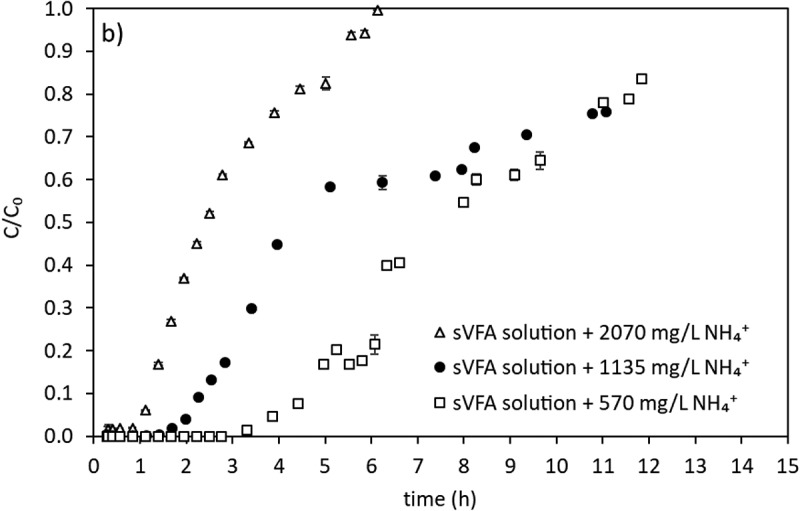
Ammonium breakthrough curves in different concentrations of sVFA solution.

In the last step of the study, in an attempt to gain a first overview, a preliminary breakthrough experiment with a solution of ammonium and sVFAs was performed with three different initial ammonium concentrations (selected based on expected ammonium concentration in the fermentation step). The number of bed volumes and number of hours processed at breakthrough point and surface concentration of the beds are used as performance indicators. Figure 10 shows the results of the breakthrough experiment for ammonium obtained with initial ammonium concentrations of 570, 1135 and 2070 mg/L at a contact time of 16.6 minutes.

In this study, the extent of NH_4_^+^removal from the VFA solution aims not to interfere with further nitrogen removal application (denitrification). Ammonium concentrations above 60 mg/L affect nitrification negatively [58]. This leads to interference with whole nitrogen removal process. In the denitrification phase, the ammonium content of the carbon source given to the environment may also enter the nitrification ammonium stream, affecting the ammonium concentration in the output stream. Hence, ammonium concentration in the ion exchange effluent should be as low as possible by continuing to use of VFAs enriched effluent in nutrient removal so as not to cause external deterioration of ammonium removal. Thereby, the point where the C/C_0_ value reached 0.01% was chosen as the breakthrough point in this study. This indicates that the column must be regenerated if the ammonium concentration at the outlet of the column is above this limit. Results showed that the breakthrough point where ammonium concentration first appeared in the effluent was reached faster at higher initial ammonium concentrations, indicating the need for further flow through studies. Increasing ammonium concentrations from 570 mg/L to 2070 mg/L resulted in sharper breakthrough curves and breakthrough for 2070 mg NH_4_^+^/L was almost immediate (3 BV or 0.8 h). However, for ammonium concentrations lower than 2070 mg/L, considerable amounts of ammonium removal, with a 99% (C/C_0_ = 0.01%) removal efficiency for about 6 BV or 1.7 h and 13 BV or 3.6 h at 1135 and 570 mg NH_4_^+^/L, respectively. Under the experimental conditions employed, the removal of ammonium was practically completed at 1–4 h depending on the initial ammonium concentration in the VFA solution. Total ammonium removed is obtained calculating the area above the breakthrough curves and dividing this area by the amount of clinoptilolite in the column gives the mass ammonium adsorbed per unit mass of zeolite for the continuous system. The surface concentration of the clinoptilolite was calculated as 17.6, 21.2, 22.7 mg NH_4_^+^-N/g clinoptilolite for 570, 1135, 2070 mg NH_4_^+^/L initial concentrations, respectively. The liquid phase concentration decreased by 32%, 20% and 15% to 17.6, 21.2 and 22.7 mg NH_4_^+^/g clinoptilolite in the sVFA solution compared to pure ammonium solution. The ammonia concentration at the effluent of column starts increasing after 3 BV (0.8 h), 6 BV (1.7 h) and 13 BV (3.6 h) for the inlet ammonium concentrations of 2070, 1135 and 570 mg/L, respectively. As the inlet concentration of ammonium decreased, breakthrough occurs at later BVs as expected. Based on the preliminary results from breakthrough, effective ammonium removal with better performance can be expected at higher contact times by either increasing the height of the bed, using a larger/bigger column structure, or decreasing the flowrate.

## 4. Conclusion

An application of the ion exchange process using a low-cost natural ion exchanger, clinoptilolite, was investigated on a laboratory scale for ammonium removal from synthetic and AD-VFA solutions. The results obtained provided valuable data for promoting biological nutrient removal processes in WWTPs as further implications with ammonium purified VFAs. Isotherm experiments showed the highest equilibrium capacity of 11.3 mg NH_4_^+^/g clinoptilolite from AD-VFA effluent with an equilibrium time of 12 hours. Ammonium removal of more than 92% from synthetic VFA and AD-VFA at a reaction time of 12 h was achieved by using a batch-mode operated clinoptilolite column. Breakthrough surface concentration per g of clinoptilolite that has been done on a mixture of synthetic VFAs and NH_4_Cl (570, 1135 and 2070 mg NH_4_^+^/L) were determined to be between 17.6 to 22.7 mg NH_4_^+^/g clinoptilolite in breakthrough studies. Although an earlier breakthrough occurs at an ammonium concentration of 2070 mg/L, larger columns or lower flow rates need to be used, as well as further studies on the regeneration of the adsorbent and the recovery of the adsorbed ammonium need to be performed. The results obtained show selective removal of ammonium with minimal loss of VFAs from VFA-containing fermentation liquid produced during digestion of nitrogen-rich feedstock.
